# Validation of Association between Breastfeeding Duration, Facial Profile, Occlusion, and Spacing: A Cross-sectional Study

**DOI:** 10.5005/jp-journals-10005-1356

**Published:** 2016-06-15

**Authors:** Shiv Shankar Agarwal, Mohit Sharma, Karan Nehra, Balakrishna Jayan, Anish Poonia, Hiteshwar Bhattal

**Affiliations:** 1 Dental Officer, Department of Orthodontics and Dentofacial Orthopedics Indian Army Dental Corps, New Delhi, India; 2 Assistant Professor, Department of Orthodontics and Dentofacial Orthopedics Armed Forces Medical College, Pune, Maharashtra, India; 3 Assistant Professor, Department of Orthodontics and Dentofacial Orthopedics Army Dental Centre (R&R), New Delhi, India; 4 Professor and Head, Department of Orthodontics and Dentofacial Orthopedics Army Dental Centre (R&R), New Delhi, India; 5 Dental Officer, Department of Pediatric and Preventive Dentistry, Army Dental Centre (R&R), New Delhi, India; 6 Dental Officer, Department of Pediatric and Preventive Dentistry, Indian Army Dental Corps, New Delhi, India

**Keywords:** Breastfeeding duration, Distoclusion, Nonnutritive sucking.

## Abstract

**Introduction:** This cross-sectional retrospective study was designed to assess the relationships among breastfeeding duration, nonnutritive sucking habits, convex facial profile, nonspaced dentition, and distoclusion in the deciduous dentition.

**Materials and methods:** A sample of 415 children (228 males, 187 females) aged 4 to 6 years from a mixed Indian population was clinically examined by two orthodontists. Information about breastfeeding duration and nonnutritive sucking habits was obtained by written questionnaire which was answered by the parents.

**Results:** Chi-square test did not indicate any significant association among breastfeeding duration, convex facial profile, and distoclusion. Statistically significant association was observed between breastfeeding duration and nonspaced dentition and also between breastfeeding duration and nonnutritive sucking habits. Nonnutritive sucking habits had a statistically significant association with distoclusion and convex facial profile (odds ratio 7.04 and 4.03 respectively). Nonnutritive sucking habits did not have a statistically significant association with nonspaced dentition.

**Conclusion:** The children breastfed < 6 months had almost twofold increased probability for developing sucking habits and nonspaced dentition, respectively, than the children who had breastfeeding > 6 months duration. It can also be hypothesized that nonnutritive sucking habits may act as a dominant variable in the relationship between breastfeeding duration and occurrence of convex facial profile and distoclusion in deciduous dentition.

**How to cite this article:** Agarwal SS, Sharma M, Nehra K, Jayan B, Poonia A, Bhattal H. Validation of Association between Breastfeeding Duration, Facial Profile, Occlusion, and Spacing: A Cross-sectional Study. Int J Clin Pediatr Dent 2016;9(2):162-166.

## INTRODUCTION

Malocclusion is not a disease and is often considered as a developmental disorder of the craniofacial complex. It may cause functional and esthetic disturbances in affected individuals.^1,2^ Environmental factors like dietary habits, nonnutritive sucking habits, such as use of pacifier, digit sucking, or bottlefeeding, and reduced duration of breastfeeding have often been implicated with various developmental disorders of the stomatognathic system.^3,4^

Breastfeeding is not mere nutrition^5^ and can be considered as a natural orthopedic appliance for the harmonious development of face.^6^ The upward and outward forces exerted by the tongue during suckling affect the growth of child’s premaxillary region and the mandibular movements stimulate mandibular growth. On the contrary, the posteriorly directed forces of buccinator during nonnutritive sucking habits restrict jaw development and disto-occlusion is frequently seen in such children.^5^

The importance of increased breastfeeding duration in preventing the development of malocclusion, enhancing the sagittal growth of mandible, and establishing a correct occlusal relationship by stimulating the facial muscles during suckling have been well documented in the literatutre.^7-11^ Few authors have demonstrated that function plays the most important role in development of facial and occlusal features and that heredity has only a secondary role,^12^ while others consider genetic influence as the major determining factor.^13^

Following a report by the World Health Organization Expert Consultation on the optimal duration of exclusive breastfeeding,^14^ on May 18, 2001, the World Health Assembly recommended minimum exclusive breastfeeding duration of 6 months as a global public health recommendation.^15^

Spaced or closed dentition has been related to several factors like mesiodistal crown diameter, intercanine, and intermolar width.^16^ The intercanine and intermolar widths in both jaws significantly increase between 3 and 5 years of age.^15^ Reduced breastfeeding duration has been variously associated with reduced intraarch transverse diameters.^5,17,18^

Taking the above factors into consideration, a study was formulated with the aim to estimate the prevalence of (1) convex facial profile, (2) distoclusion of deciduous second molars, (3) nonspaced/closed dentition, and (4) the association of above mentioned parameters with the duration of breastfeeding and nonnutritive sucking habits in children who were in their deciduous dentition stage.

## MATERIALS AND METHODS

This cross-sectional study was conducted in the Division of Orthodontics and Dentofacial Orthopedics, Armed forces medical college, Pune, India. A written questionnaire was distributed to 650 parents of children who were in their deciduous dentition (aged 4-6 years) studying in two primary schools in Pune, Maharashtra. The questionnaire was framed based on the inclusion and exclusion criteria of the study and also contained complete information about age, gender, height, and weight of the children. Information about mother’s health during pregnancy and type of delivery was also gathered and only full-term and normally born children were included in the study. The inclusion criteria formulated for the study was as under:

- Age 4 to 6 years
- A student studying at one of the two chosen schools for the study
- Normal number, size, and shape of teeth present
- Absence of root stumps and teeth with poor prognosis
- Unerupted or partially erupted permanent first molars not in occlusion
- Parental completion of the questionnaire about the child’s habits, and no oral or systemic condition which may affect bone metabolism
  The exclusion criteria formulated was as under:
- Presence of any local or systemic disease in the child which may affect bone metabolism
- Any anomaly in the number, shape, or size of the teeth
- Presence of rampant caries and teeth with poor prognosis
- Fully erupted permanent first molars
- Parental refusal to fill the written questionnaire.

Based on the above-mentioned criteria, 415 children (228 males and 187 females) were finally selected for the study (Table 1). The children were divided into two groups: Group 1 (children exclusively breastfed ≥ 6 months (n = 158)) and group 2 (children exclusively breastfed ≤ 6 months (n = 257)) (Table 1). A retrospective investigation was made for the length of time that children were exclusively breastfed in the questionnaire. Information on nonnutritive sucking habits was also included in the questionnaire.

**Table 1:** **Table 1:** The age distribution of cases studied

*Age (years)*		*Breastfeeding* *≤ 6 months*		*Breastfeeding* *≥ 6 months*		*Total*	
4		0		1 (0.4)		1 (0.2)	
5		61 (38.6)		80 (31.1)		141 (34.0)	
6		97 (61.4)		176 (68.5)		273 (65.8)	
Total		158 (100.0)		257 (100.0)		415 (100.0)	

Values are n (% of cases)

The clinical examination was performed by two orthodontists based on our study protocol. The interexaminer reliability was determined by means of Kappa coefficient which was 0.858 and p-value was 0.001 (highly significant). The occlusal relationships were examined by direct visual inspection with the teeth in centric occlusion. The sagittal interarch relationship was recorded according to the distal relationship of the maxillary and mandibular primary second molars as classified by Baume:^19^

- Flush terminal plane: Forming a straight line.
- Distal step terminal plane: Forming a distal step to the mandible.
- Mesial step terminal plane: Forming a mesial step to the mandible.

Facial profiles were clinically evaluated according to the following criteria^20^ and classified as straight, convex or straight:

- Straight: Class I facial pattern with no sagittal discrepancy between jaws.
- Convex: Class II facial pattern with mandibular retrusion, maxillary protrusion, or both.
- Concave: Class III facial pattern with maxillary retrusion, mandibular protrusion, or both.

Similarly, the dentition was marked as spaced or nonspaced (closed) dentition depending on the presence or absence of physiologic spaces in the dentition.

Data were accumulated from the questionnaires as well as findings of the clinical examination and recorded in excel sheets. A chi-square test (p < 0.05) was performed to verify associations between (1) breastfeeding duration and prevalence of nonnutritive sucking habits, (2) breastfeeding duration and convex facial profile, (3) breastfeeding duration and distoclusion of deciduous second molars, (4) breastfeeding duration and nonspaced dentition, and (5) nonnutritive sucking habits and all the above mentioned parameters. In order to measure the strength of the associations tested, an odds ratio (OR) was calculated.

## RESULTS

The prevalence of distoclusion, convex facial profile, and nonspaced dentition was 18.6, 16.1, and 40.2% respectively. The frequency of breastfeeding for < 6 months duration was 158 (38.1%), and the frequency of breastfeeding > 6 months duration was 257 (61.9%). Nonnutritive sucking habits were observed in 63 (15.2%) of the children studied (Table 2).

**Graph 1:**
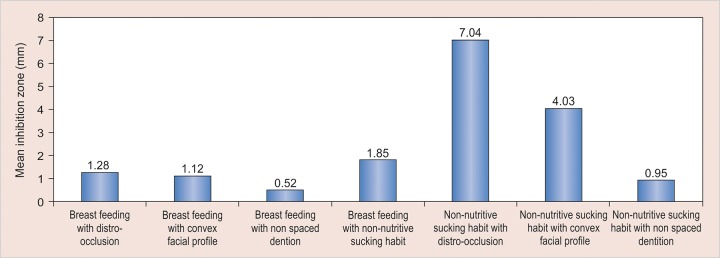
Associations tested

On statistical analysis (chi-square test), no significant association was observed between breastfeeding duration and distoclusion. In addition, there was no statistically significant association between breastfeeding duration and convex facial profile. However, the association between breastfeeding duration and nonspaced dentition and also between breastfeeding duration and nonnutritive sucking habits was statistically significant. The OR assessment showed that children breastfed for less than 6 months had almost twofold (OR 1.85 and 1.92 respectively) increased probability for developing sucking habits and nonspaced dentition, respectively, than the children who had breastfeeding for more than 6 months duration (Tables 2 and 3) (Graph 1).

**Table 2:** **Table 2:** Frequency of distoclusion, convex facial profile, nonnutritive sucking habit, and non-spaced dentition according to breastfeeding duration

		*Breastfeeding* *≤ 6 months*		*Breastfeeding* *≥ 6 months*		*Total*	
Distoclusion		33 (20.9)		44 (17.1)		77 (18.6)	
Nondistoclusion		125 (79.1)		213 (82.9)		338 (81.4)	
Convex facial profile		27 (17.1)		40 (15.6)		67 (16.1)	
Nonconvex facial profile		131 (82.9)		217 (84.4)		348 (83.9)	
Nonnutritive sucking habit		32 (20.3)		31 (12.1)		63 (15.2)	
No nonnutritive sucking habit		126 (79.7)		226 (87.9)		352 (84.8)	
Spaced dentition		79 (50.0)		169 (65.8)		248 (59.8)	
Nonspaced dentition		79 (50.0)		88 (34.2)		167 (40.2)	
Total		158 (100.0)		257 (100.0)		415 (100.0)	

Values are n (% of cases)

**Table 3:** **Table 3:** Associations tested

*Associations*		*Chi-square* *value*		*p-value*		*OR*		*95% CI* *for OR*	
Breastfeeding with distoclusion		0.918		0.338		1.28		0.77-2.11	
Breastfeeding with convex facial profile		0.168		0.682		1.12		0.66-1.91	
Breastfeeding with nonspaced dentition		10.104		0.001		1.92		1.28-2.88	
Breastfeeding with nonnutritive sucking habit		5.098		0.024		1.85		1.08-3.18	
Nonnutritive sucking habit with distoclusion		51.087		0.001		7.04		3.93-12.64	
Nonnutritive sucking habit with convex facial profile		22.750		0.001		4.03		2.20-7.35	
Nonnutritive sucking habit with nonspaced dentition		0.033		0.857		0.95		0.55-1.64	

p-value < 0.05 indicates significant associations; OR: Odds ratio; CI: Confidence interval

**Table 4:** **Table 4:** Frequency of distoclusion, convex facial profile, and nonspaced dentition according to nonnutritive sucking habits

		*Nonnutritive* *sucking habit*		*No nonnutritive* *sucking habit*		*Total*	
Distoclusion		32 (50.8)		45 (12.8)		77 (18.6)	
No distoclusion		31 (49.2)		307 (87.2)		338 (81.4)	
Convex facial profile		23 (36.5)		44 (12.5)		67 (16.1)
Nonconvex facial profile		40 (63.5)		308 (87.5)		348 (83.9)	
Spaced dentition		37 (58.7)		211 (59.9)		248 (59.8)	
Nonspaced dentition		26 (41.3)		141 (40.1)		167 (40.2)	
Total		63 (100.0)		352 (100.0)		415 (100.0)	

Values are n (% of cases)

Nonnutritive sucking habit had a statistically significant association with distoclusion and convex facial profile (OR 7.04 and 4.03 respectively). Nonnutritive sucking habit did not have statistically significant association with nonspaced dentition (Tables 3 and 4) (Graph 1).

## DISCUSSION

The literature hosts an ample amount of controversy on whether environmental influences like reduced infantile breastfeeding practices can prevent normal development of orofacial structures including retarded anteroposterior growth of mandible and distoclusion of the deciduous dentition^7-12^ or genetics plays a larger role.^13,21^ The results of our study do not support the hypothesis that the environmental influence of breastfeeding plays a major role in mandibular development since statistically significant association was not obtained between reduced duration of breastfeeding and convex profile in our study. These findings support those of Luz et al^22^ who had conducted a similar study on Brazilian children. However, in their study, the children were of higher age group (5-11 years). These findings contradict with a few authors who consider breastfeeding as a stimulus for mandibular development and establishing correct intermaxillary relationships.^8,9,23,24^

Bishara et al^25^ in a longitudinal study observed that cases with distoclusion in the primary dentition resulted in class II molar relation in the permanent dentition which did not self-correct with growth of the child and concluded that flush or mesial step was a more favorable molar relation in the deciduous dentition as it reduces the chances of distoclusion in the permanent dentition. In another study,^26^ an association between breastfeeding duration of less than 6 months and higher occurrence of Angle class II malocclusion were observed but the age group in their study was higher than ours (12-15 years). In our study, higher frequency of children who were breastfed ≤6 months had distal step terminal plane (20.9%) than those breastfed ≤6 months, but this association was not statistically significant.

The presence of physiological spaces in the deciduous dentition and its importance in the development of correct intermaxillary relation has often been emphasized in the literature.^5,16-18^ According to a study by Bishara et al,^17^ the maxillary and mandibular transverse diameters increase significantly between 3 and 5 years of age. Therefore, the subjects in our study were limited to less than 6 years presuming that any environmental stimulus at this stage may affect the occurrence of physiological spaces. Lopez Del Valle^5^ observed a higher frequency of closed dentition in their study (31%) and attributed this space deficiency to reduced duration of breastfeeding. In our study, a higher frequency of nonspaced dentition was found in children breastfed ≤6 months (50%) than in the other group (34.2%), and this association was statistically significant. However, no statistically significant association was observed between nonspaced dentition and nonnutritive sucking habits in our study.

A statistically significant association was observed in our study between breastfeeding duration and nonnutritive sucking habits and also between nonnutritive sucking habits and distoclusion and convex facial profile (OR 7.04 and 4.03 respectively). These findings were similar to those of Mossey^13^ and Praetzel and Abrahao^26^ who consider facial growth patterns to be genetically determined against the dentoalveolar structures which are more influenced by external environment factors like breastfeeding. Our findings also point to a hypothesis that nonnutritive sucking habits may act as a dominant variable in the relationship between breastfeeding duration and convex facial profile and distoclusion of the dentition. The OR assessment in our study showed that children breastfed for less than 6 months had almost twofold increased probability for developing sucking habits and nonspaced dentition respectively, than the children who had breastfeeding for more than 6 months duration.

## CONCLUSION

From this study, the following conclusions can be made:

- The occurrence of convex facial profile and distoclusion was not associated with breastfeeding duration but was associated with nonnutritive sucking habits.
- The presence of nonspaced dentition was associated with reduced breastfeeding duration but not with nonnutritive sucking habits.
- A significant association was observed between reduced duration of breastfeeding and higher prevalence of nonnutritive sucking habits.
- Nonnutritive sucking habits may be hypothesized as a dominant variable in the relationship between breastfeeding duration and convex facial profile and distoclusion of the dentition.
- Further longitudinal studies with a larger sample size are required to be conducted to further enhance our knowledge on these issues.
